# The defensive role of foliar endophytic fungi for a South American tree

**DOI:** 10.1093/aobpla/plw050

**Published:** 2016-08-02

**Authors:** Marcia González-Teuber

**Affiliations:** Departamento de Biología, Universidad de La Serena, Casilla 554, La Serena, Chile

**Keywords:** *Embothrium coccineum*, endophyte diversity, fungal endophytes, inhibitory effects, pathogens, plant protection

## Abstract

Fungal endophytes colonize living internal plant tissues without causing any visible symptoms of disease. Endophytic fungi associated with healthy leaves may play an important role in the protection of hosts against herbivores and pathogens. In this study, the diversity of foliar endophytic fungi (FEF) of the southern temperate tree *Embothrium coccineum* (Proteaceae), as well as their role in plant protection in nature was determined. Fungal endophytes were isolated from 40 asymptomatic leaves by the culture method for molecular identification of the 18S rRNA gene. A relationship between FEF frequency and plant protection was evaluated in juveniles of *E. coccineum*. Fungal endophyte frequency was estimated using real-time PCR analyses to determine endophyte DNA content per plant. A total of 178 fungal isolates were identified, with sequence data revealing 34 different operational taxonomic units (OTUs). A few common taxa dominated the fungal endophyte community, whereas most taxa qualified as rare. A significant positive correlation between plant protection (evaluated in terms of percentage of leaf damage) and FEF frequency was found. Furthermore, in vitro confrontation assays indicated that FEF were able to inhibit the growth of fungal pathogens. The data showed a relatively high diversity of fungal endophytes associated with leaves of E. *coccineum*, and suggest a positive relationship between fungal endophyte frequencies in leaves and host protection in nature.

## Abstract (precis)

Endophytic fungi associated with healthy leaves of the South American tree *Embothrium coccineum* (Proteaceae) appear to play an important role in host protection in nature. Results showed that a few common taxa dominated the fungal endophyte community in leaves of *E. coccineum*, and demonstrated that higher infection rates of the dominant endophyte genera correlated with lower levels of leaf damage in the host plant. Furthermore, *in vitro* confrontation assays indicated that foliar endophytic fundi were able to successfully reduce the growth of fungal pathogens. These results provide evidence that colonization by multiple foliar endophytic fungi confers important benefits to host plants in terms of resistance to natural enemies in the field.

## Introduction

Plants interact with a variety of microbes in their roots, stems and leaves (Partida-Martinez and Heil 2011). Fungal endophytes frequently occur in aerial plant structures, living inter-cellularly in leaf and stem tissue (Clay 1990) for at least part of their life cycle without causing any apparent sign of disease (Wilson 1995). These fungal associations are common in angiosperms, but have been particularly found and described for grasses (Poaceae family) (Saikkonen *et al.* 2004). The associations between grasses and ‘type I’ endophytic fungi of the Clavicipitaceae family have been well documented, as the latter colonize the host systemically, and are vertically transmitted in a classic example of mutualism. Non-clavicipitaceous ‘type II’ endophytes are highly diverse, and in contrast to type I, are non-systemic and mostly horizontally transmitted (Rodriguez *et al.* 2009). Despite the fact that type II endophytes are ubiquitous, having been found in all plant species to date (Arnold *et al.* 2000; Arnold and Herre 2003; Albrectsen *et al.* 2010; Gazis and Chaverri 2010), they are far less well studied and their ecological roles are not yet fully understood.

Increased resistance to pathogens and/or herbivores may be a consequence of plant colonization by fungal endophytes (Partida-Martinez and Heil 2011). Host protection from natural enemies provided by clavicipitaceous endophytes, as well as their mechanisms of action, has been extensively studied (Clay 1988; Wilkinson *et al.* 2000; Cheplick and Faeth 2009). Much less is known about the role of non-clavicipitaceous endophytes in this regard, although there is evidence that type II also contribute to plant protection from pathogens (Arnold *et al.* 2003; Adame-Álvarez *et al.* 2014) and herbivores (Van Bael *et al.* 2009; Bittleston *et al.* 2011; Estrada *et al.* 2015). Several studies have reported high infection rates by type II FEF in plants (Arnold *et al.* 2000; Arnold and Herre 2003; Gazis and Chaverri 2010). Since infection intensity (frequency of FEF in host tissues) by type II endophytes is not systematic, infection intensity may be an important factor determining plant resistance to enemies. Accordingly, an earlier study showed that a higher infection frequency of the dominant fungal endophyte in oak trees was negatively correlated with the performance of a leaf mining herbivore (Preszler *et al.* 1996).

**Figure 1.**
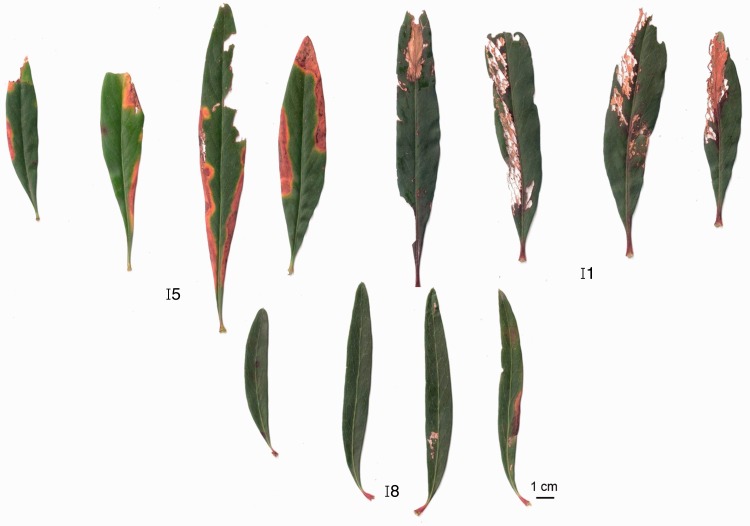
Differences in leaf damage among juveniles of *Embothrium coccineum* in the field. Four leaves from three individuals (I: individuals 1, 5 and 8) are shown.

*Embothrium coccineum* (Proteaceae) is a small tree endemic to South American temperate forests commonly occurring in open sites (Díaz and Armesto 2007). Embothrium *coccineum* suffers relatively high levels of damage by pathogens and/or herbivores under natural conditions (Baldini and Pancel 2002; Salgado-Luarte 2010; approximately 30% of leaf damage, personal observations). Interestingly, marked variations in the amount of leaf damage in juvenile plants of this species are evident; whereas leaves of some juveniles show significant damage, no signs of damage are evident in others (Fig. 1, **Supporting Information—Fig. s1**). This raises the question of whether higher FEF frequencies may relate to improved plant protection in nature. In light of the above, this study assessed the following questions: (1) How diverse is the community of FEF associated with asymptomatic leaves of *E. coccineum*? (2) Is there a relationship between FEF frequency and host plant resistance in nature? (3) Do foliar endophytic fungi inhibit the growth of fungal pathogens *in vitro*?

## Methods

### Study site

The study site (Anticura) was situated in mature temperate rainforest at Puyehue National Park (−40.65S, −72.18W; 350–400 m a.s.l.), in the western foothills of the Andes in southern Chile. Mean annual precipitation at the site is 2800 mm, with a mean temperature of 9.8 °C (Dorsch 2003). Old-growth lowland forest in this region is composed of broad-leaved evergreen trees (Lusk and Del Pozo 2002). *Embothrium coccineum* (Chilean firetree, Proteaceae) is one of the dominant species in the rainforest, and is susceptible to damage by herbivores and pathogens at the juvenile stage (estimated 30% under natural conditions). *Embothrium* *coccineum* shows fast growth in comparison to other native species (Escobar *et al.*, 2006).

### Isolation of fungal endophytes

Ten *E. coccineum* individuals were randomly selected for leaf collection, undertaken in March 2014. All plants were juveniles between 1.5 and 2.0 m in height, at similar developmental stage (plant diameter and number of leaves). Four healthy (0% damage) mature leaves per individual were collected and immediately transported to the lab for further endophyte isolation and molecular characterization. Leaves were disinfected under laboratory conditions with different washes of ethanol (70%), sodium hypochlorite (1%) and sterilized water (according to Arnold et al. 2001, 2003). The success of the surface sterilization method was confirmed by the absence of any microbial growth on PDA (potato-dextrose-agar) (Phyto Technology Laboratories) agar plates from the plating of last washing water. Small sections of sterilized leaves (2–3 mm) were subsequently cultivated on PDA petri dishes plates. Plates were then incubated at room temperature for 2–3 weeks. After that time, emerging colonies were subcultured to obtain pure isolates. Pure isolates were grown on PDA plates (Phyto Technology Laboratories) at room temperature for one month for further DNA extraction and molecular identification.

### Molecular characterization of endophytes

Genomic DNA was extracted from the mycelial mat using a modified method described by Nicholson *et al.* (2001). Fresh mycelium was ground on Mini-BeadBeater-16 (BioSpec, USA). Ground micelyum was suspended in extraction buffer (10 mM Tris buffer pH 8.0, 10 mM EDTA, 0.5% SDS, NaCl 250 mM). To this aqueous solution, phenol:chloroform:isoamyl alcohol (25:24:1) was added and mixed slowly for 3 min. The phases were separated by centrifugation at 13.000 rpm for 10 min at room temperature. Traces of phenol were removed by treating the aqueous layer with chloroform:isoamyl alcohol (24:1) (this step was repeated twice). Phases were separated as before. DNA was precipitated from the aqueous phase with 2.0 volumes of isopropanol. The DNA was recovered by centrifugation at 10 000 rpm for 15 min at 4 °C. The pellet was then washed with 70% ethanol and resuspended in molecular biology grade water (Mo Bio Laboratories, Inc). Species identification of endophytic fungi was performed using the primers ITS1-F (CTTGGTCATTTAGAGGAAGTAA) (Gardes and Bruns 1993) and ITS4 (TCCTCCGCTTATTGATATGC) (White *et al.* 1990). Amplification of the ITS (internal transcribed spacers) region (around 680 kbp) was conducted using 50 µL of PCR reaction mixtures, each containing 7 µL of total fungal genomic DNA, 1 µL of each primer (at a concentration of 10 µM for each primer), 27.5 µL of SapphireAmp Fast PCR Master Mix (Takara) and 13.5 µL of sterilized water. PCR was performed in a Techne TC-5000 Thermal Cycler (Fisher Scientific) with the following program: 94 °C for 3 min, followed by 35 cycles of denaturation at 94 °C for 1 min, annealing at 54 °C for 30 s and primer extension at 72 °C for 1 min, completed with a final extension at 72 °C for 7 min. PCR products were sent to Macrogen (South Korea) for purification and sequencing. Sequences were assembled using SeqTrace software. Consensus sequences were used for BLAST searches at the NCBI (http://www.ncbi.nlm.nih.gov).

### Relationship between endophytic frequency and leaf damage under natural conditions

Twenty *E. coccineum* juveniles were selected in the field in March 2014, and three leaves from each were randomly collected for determination of fungal endophyte DNA content (infection frequencies) and leaf damage (percentage of leaf damage was considered as a proxy for plant protection in nature). To calculate the percentage of leaf damage, leaves from each plant were scanned and stored as digital image files. Total leaf area (TA) and damage area (DA) were determined for each sample using the program ImageJ (http://imagej.nih.gov/ij/). Average TA and DA for the three leaves were used to derive per plant values. Given the difficulty of differentiating pathogen from herbivore damage, they are referred hereafter collectively as damage by natural enemies.

**Figure 2.**
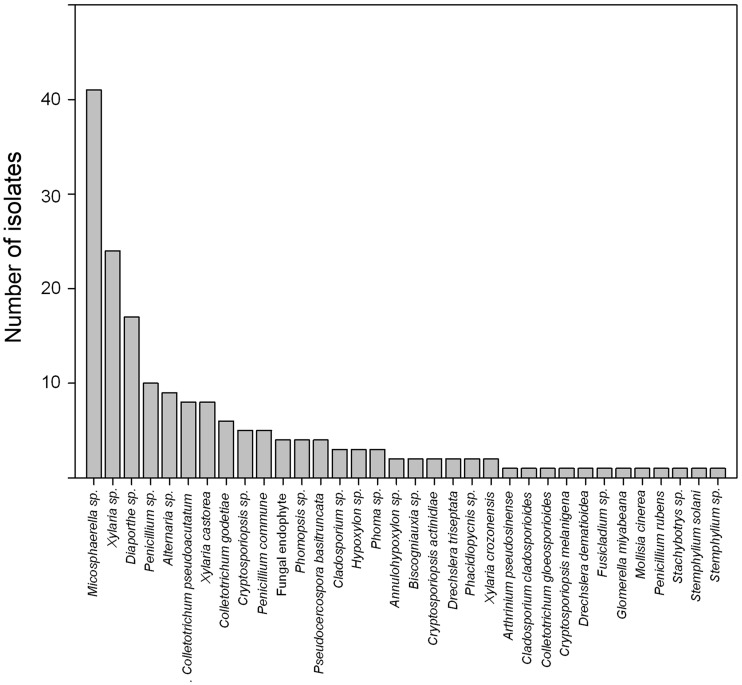
Frequency of endophyte taxa isolated from asymptomatic leaves of *Embothrium coccineum*.

The FEF DNA content was only evaluated for those endophyte species that exhibited the highest frequencies in *E. coccineum*, and accounted for 70% of the fungal community (according to Fig. 2): *Mycosphaerella sp.*, *Diaporthe sp.*, *Xylaria sp.* and *Penicillum sp.* Total DNA (2 g per individual plant, according to Lambertini *et al*. 2008) was extracted from disinfected leaves (three leaves were collected and pooled per plant) of the same 20 juveniles mentioned above and subjected to real-time PCR (q-PCR) assays using primers and probes, which were specifically designed to recognize the four FEF genera (*Mycosphaerella*, *Diaporthe*, *Penicillium* and *Xylaria*) (**See Supporting Information—Table S1**). Some of the leaves showed a high degree of damage, potentially by pathogenic fungi, and the use of universal fungal primers for q-PCR analyses was, therefore, inappropriate, since this would also result in the amplification of fungal pathogens. Primers and probes were designed using Primer3 (Koressaar and Remm 2007; Untergasser *et al.* 2012). Primers were commercially synthesized in Macrogen (South Korea). q-PCR analyses were performed in a Stratagene Mx3000P (Thermo Fisher Scientific), using the kit Brillant II QPCR Master mix (Agilent Technologies) and the primers and probes TaqMan with fluorophores FAM-TAMRA. A standard curve based on threshold cycle (Ct) was constructed for each fungal endophyte. Samples (100 ng DNA per plant) were then run in triplicate in each plate. PCR cycling parameters were 95 °C for 10 min, 40 cycles at 95 °C for 15 s, 55 °C for 30 s and 72 °C for 1 min. The cycle at which a sample’s signal exceeds the threshold, the CT value, was used to calculate total amplified DNA, using a formula obtained from the slope of the regression line from the standard curve. Potential differences in ribosomal RNA gene copies among endophytes might relate to an overestimation of total amplified DNA observed for each fungus.

Correlations between the percentage of leaf damage and FEF frequency (fungal endophyte DNA content per plant in ng) were tested with the non-parametric Spearman´s rho test. Correlations were done for each endophytic fungus (*Mycosphaerella*, *Diaporthe*, *Penicillium* and *Xylaria*), and for the four endophytic fungi taken as a whole. The latter was calculated by adding the DNA contents of the four dominant endophytes. All analyses were performed in Statistica 7.0 software (StatSoft, Inc.).

### Confrontation assays

For the confrontation assays, the four most abundant endophytes were confronted against three common fungal pathogens of endemic woody plants in Chile (*Botrytis cinerea*, *Fusarium oxysporum* and *Ceratocystis pilifera*) (Baldini and Pancel 2002). Fungal pathogens were kindly provided by the Laboratorio de Química de Productos Naturales, Universidad de Concepción, Chile. Endophytes and pathogenic fungi were co-cultured on 90 mm petri dishes containing PDA (potato dextrose agar) at 21 °C over a period of 11 days. Sections (1 cm^2^) of each fungal mycelium were placed 4 cm apart on a fresh potato dextrose agar plate. Pathogens cultivated in isolation served as controls. Microbial growth was determined by measuring the diameter of the colonies every day over the 11-day period, and comparing colony sizes in the confrontation situation to that of the controls. The percentage of growth inhibition (GI) was calculated with the following formula: GI = ((A−B)/A) * 100, where *A* is the radial diameter of the control pathogen; *B* is the radial diameter of test pathogen.

In order to evaluate potential inhibitory or stimulatory effects on growth among endophytes themselves, the four endophytes (*Mycosphaerella sp.*, *Diaporthe sp.*, *Penicillium sp.* and *Xylaria sp.*, named confronted endophytes) were confronted against themselves (named tested endophytes). Assays were carried out as described above, again with endophytes cultivated in isolation used as controls. The percentage of growth was evaluated over a 5-day period using the same process described earlier. Whereas positive values would indicate inhibitive effects on growth of confronted endophytes on tested endophytes, negative values would by contrast suggest a stimulative effect.

## Results

### Diversity and composition of fungal endophytes

Of the 10 *E. coccineum* juveniles screened in this study, a total of 178 fungal isolates were purified into individual cultures. All plants (incidence = 100%), but not all leaves (incidence = 82.5%) contained fungal endophytes. A total of 34 OTUs were identified (Table 1). The distribution of fungal isolates revealed a few common taxa, and many rare taxa (Fig. 2). The endophytic fungal community was dominated by the genera *Mycosphaerella sp*. (23%), *Xylaria sp*. (18%) and *Diaporthe sp*. (10%). *Penicillium* and *Colletotrichum* genera occurred at frequencies lower than 10%, whereas other genera were found in rare instances, with frequencies between 1% and 5%. Total species richness was 34, with an evenness of 0.80. The Simpson index suggested a relatively high diversity of fungal endophytes associated with *E. coccineum*. Simpson diversity was 0.90 (with a range of 0–1, with 1 representing higher sample diversity).

**Table 1.** Best BLAST matches for isolated fungal endophyte OTUs collected from asymptomatic leaves of *Embothrium coccineum* in the Valdivian rainforest.

Endophytic fungi	Accession number	Identity (%)
*Alternaria sp.*	KP985749.1	99
*Annulohypoxylon sp.*	JQ327866.1	99
*Arthrinium sp.*	NR_121559.1	93
*Biscogniauxia sp.*	JN225898.1	97
*Cladosporium cladosporioides*	AF455442.1	98
*Cladosporium sp.*	KF367501.1	99
*Colletotrichum gloeosporioides*	AJ301972.1	100
*Colletotrichum godetiae*	KC860043.1	97
*Colletotrichum pseudoacutatum*	NR_111756.1	99
*Cryptosporiopsis actinidiae*	KF727420.1	99
*Cryptosporiopsis melanigena*	AF141196.1	98
*Cryptosporiopsis sp.*	JF288555.1	99
*Diaporthe sp.*	JN225920.1	98
*Drechslera dematioidea*	JN712465.1	99
*Drechslera triseptata*	AF163059.1	99
Fungal endophyte	EU686153.1	96
*Fusicladium sp.*	GU446639.1	98
*Glomerella miyabeana*	JN943455.1	99
*Hypoxylon sp.*	KJ406985.1	89
*Mollisia cinerea*	DQ491498.1	98
*Mycosphaerella sp.*	JN225927.1	100
*Penicillium commune*	KR012904.1	99
*Penicillium rubens*	LC015689.1	99
*Penicillium sp.*	KT264644.1	99
*Phacidiopycnis sp.*	JN944643.1	99
*Phoma sp.*	JN225888.1	100
*Phomopsis sp.*	AY518680.1	98
*Pseudocercospora basitruncata*	DQ267600.1	99
*Stachybotrys sp.*	KR081400.1	100
*Stemphylium solani*	AF203448.1	98
*Stemphylium sp.*	JX164072.1	99
*Xylaria castorea*	JN225908.1	99
*Xylaria crozonensis*	GU324748.1	99
*Xylaria sp.*	JN225909.1	100

### Protection of fungal endophytes in nature and in *vitro*

**Figure 3.**
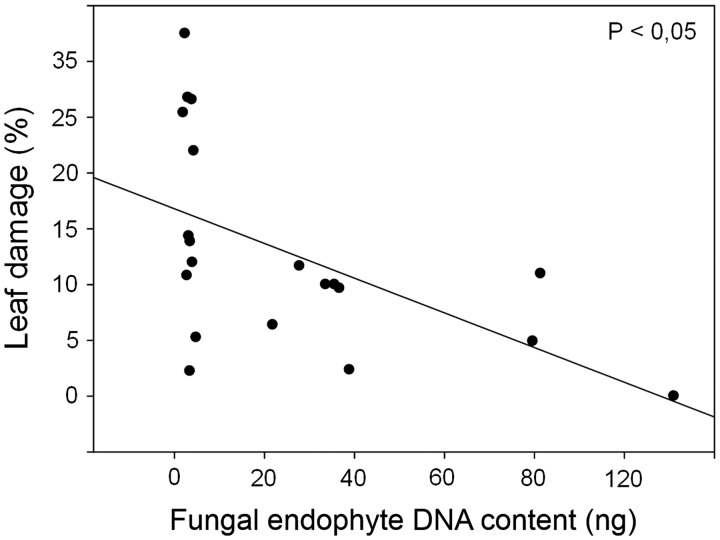
Correlation between fungal endophyte DNA content (per 100 ng of plant DNA) and the percentage of leaf damage in twenty individuals of *Embothrium coccineum*.

A significant negative correlation between FEF DNA content and percentage of leaf damage was found when frequencies of the four endophytic fungi were summed (*r* = −0.53, *P* = 0.0156) (Fig. 3). Nevertheless, no significant relationship between the DNA amount of any of the four fungal endophytes and the % of leaf damage was detected (*Mycosphaerella sp.*: *r* = −0.34, *P* = 0.135; *Diaporthe sp*.: *r* = −0.36, *P* = 0.113; *Xylaria sp*.: *r* = −0.35, *P* = 0.119; *Penicillium* sp.; *r* = −0.27, *P* = 0.232).

All three pathogen species demonstrated significant reductions in growth relative to experimental controls when exposed to endophytes; *Penicillium* showed the strongest inhibitory effects against the three pathogens, whereas the FEF *Diaporthe sp*., *Mycosphaerella sp*. and *Xylaria sp*. showed weaker inhibitory effects (Table 2). Of the three pathogens, inhibitory effects by all endophytes were most pronounced in *Fusarium sp*. and *Ceratoystis pilifera*, with *Botrytis cinerea* demonstrating relatively lower levels of inhibition. Tests for effects on growth among endophytes themselves demonstrated an overall positive effect between the most dominant endophytes (Table 3); *Mycosphaerella* was the only endophyte that demonstrated inhibitory effects, although negligible.

**Table 2:** Inhibition effects (%) of the four most abundant fungal endophytes isolated from *E.* *coccineum* against the fungal pathogens *Fusarium oxysporum*, *Ceratocystis pirifera* and *Botrytis cinerea* in confrontation assays in Petri dishes. Values indicate the mean ± SE (standard error) (*N* = 3).

	*Fusarium oxysporum*	*Ceratocystis pilifera*	*Botrytis cinerea*
*Xylaria sp*.	17.7 ± 1.96	7.6 ± 0.49	4.05 ± 0.65
*Penicillium sp*.	28.7 ± 2.08	31.8 ± 3.58	17.8 ± 3.52
*Diaporthe sp*.	30.4 ± 1.70	33.8 ± 6.69	27.7 ± 1.62
*Mycosphaerella sp*.	20.0 ± 0.04	22.5 ± 2.06	8.1 ± 2.04

**Table 3.** Growth (%) results from confrontation assays of fungal endophytes themselves. Positive values in brackets indicate growth inhibition effects of confronted endophytes on tested endophytes. Negative values in brackets indicate a positive growth effect of confronted endophytes on tested endophytes. Values indicate the mean ± SE (standard error) (*N* = 3).

	**Tested endophytes**			
Confronted endophytes	*Xylaria sp*.	*Penicillium sp*.	*Diaporthe sp*.	*Mycosphaerella sp*.
*Xylaria sp*.		**(-)** 7.8 ± 5.3	**(-)** 10.9 ± 4.0	**(-)** 9.1 ± 3.6
*Penicillium sp*.	**(-)** 3.9 ± 3.4		**(-)** 7.1 ± 3.5	**(-)** 3.1 ± 1.4
*Diaporthe sp*.	**(-)** 9.1 ± 2.6	**(+)** 0.2 ± 0.7		**(+)** 3.0 ± 3.4
*Mycosphaerella sp*.	**(+)** 2.5 ± 1.9	0.0 ± 2.9	**(+)** 3.6 ± 4.1	

## Discussion

The main aims of this study were to investigate the diversity of foliar endophytic fungi associated with *E. coccineum*, and to determine whether correlations between endophyte infection frequencies and plant resistance occur. The present study provides evidence that infection by the dominant endophyte community confer host protection from natural enemies in the field.

The endophytic fungal community associated with *E. coccineum* was represented primarily by four genera, *Mycosphaerella*, *Xylaria*, *Diaporthe* and *Penicillium*, which collectively accounted for 70% of the culturable community. Culture-based methods may underestimate diversity and misrepresent taxonomic composition of endophyte communities (Arnold 2007; Hyde and Soytong 2008) in comparison with culture-independent approaches, such as DNA cloning (Guo *et al.* 2001) or PCR product pyrosequencing (Nilsson *et al.* 2009). Despite the fact that culture-independent approaches demonstrate certain advantages over the culture-based techniques, including the capacity to detect unculturable species or species with low abundances (Hyde and Soytong 2008), comparative studies of both methods indicate that many of the proportionally dominant microbial taxa identified by culture-independent approaches are accurately represented by the culture method (see Pitkäranta *et al.*, 2011; Jackson *et al.*, 2013; Bodenhausen *et al.*, 2013).

Data here support earlier findings that a relatively small number of endophyte species dominate the fungal community, and that most taxa, with few isolations, qualify as rare (Arnold *et al.* 2001; Gazis and Chaverri 2010). Endophyte genera *Xylaria, Mycosphaerella* and *Penicillium* are commonly isolated from leaf tissues of different plant species at a range of latitudes (Arnold and Lutzoni 2007; Gazis and Chaverri 2010; Douanla-Meli *et al.* 2013), which indicates their ability to colonize a broad host range. Despite the fact that FEF diversity associated with *E. coccineum* was probably underestimated, OTU richness found here appears similar to that reported for other woody plants. For example, 58 OTUs were isolated from leaves and stems of the Peruvian tropical tree *Hevea brasiliensis* (Gazis and Chaverri 2010); 242 and 259 FEF morphospecies were recovered from the neotropical trees *Heisteria concinna* and *Ouratea lucens*, respectively (Arnold *et al.* 2001), and 89 OTUs were obtained from photosynthetic tissue of four woody desert plants (Massimo *et al.* 2015).

Host plant benefits conferred by FEF are predominantly reported in studies investigating endophyte effects on plant resistance based on experimental applications of endophytic fungi. These studies demonstrate that FEF are able to effectively protect hosts from pathogen and herbivore attack (Arnold *et al.* 2003; Van Bael *et al.* 2009; Bittleston *et al.* 2011). For example, in the tropical tree *Theobroma cacao* (Malvaceae), a combination of the seven most dominant endophytes (previously isolated from the host plant) effectively prevented host pathogen damage (Arnold *et al.* 2003). Furthermore, Van Bael *et al.* (2009) demonstrated that high endophyte frequencies in leaves of the tropical vine *Merremia umbellate* had a negative effect on herbivore fecundity relative to endophyte-free plants. Using q-PCR analyses, the present study tested the relationship between endophyte infection frequencies and host plant protection in nature. Results showed that higher infection rates of the dominant endophyte genera correlate with lower levels of leaf damage in *E. coccineum*. q-PCR analyses were unable to detect dominant endophyte DNA in several collected samples. The endophyte incidence in leaves of *E. ccocineum* was of 82.5%, which might help to explain this result. An incidence of foliar endophytic fungi of less than 100% appears to be not atypical (see Gazis and Chaverri 2010).

A significant negative correlation between FEF frequency and plant protection was found for the four endophyte genera as a whole, although no correlation between individual endophyte genera and leaf damage was evident. The latter result strongly supports the ‘multiple defender effect’ hypothesis (McKeon *et al.* 2012), which states that the presence of multiple mutualists act to produce protective benefits synergistically or additively. Synergistic protective effects by fungal endophytes have been described (Gazis and Chaverri 2015); for example, the abundance, as opposed to just the presence, of competitive endophyte strains and species has been shown to confer protective benefits in wild *Hevea* trees (Gazis and Chaverri 2015). Furthermore, combined effects on growth of arbuscular mycorrhizal fungi and fungal endophytes were found to be additive in the grass *Elymus hystrix* (Larimer et al. 2012). Results here provide further evidence that cooperation among endophytes leads to enhanced protective effects for the host plant.

Endophytes are known to produce a large number of specific toxins (Schulz *et al.* 2002; Strobel and Daisy 2003; Mousa and Raizada 2013) that directly affect the growth and performance of herbivores and pathogens. Furthermore, by changing leaf chemical characteristics, it has been shown that FEF negatively influence herbivore preferences for *Cucumis sativus* (Estrada *et al.* 2013). Confrontation assays in Petri dishes showed that FEF investigated in this study were able to effectively inhibit the growth of several woody plant pathogens, which is likely a result of active inhibition, due to the presence of an inhibition zone. *Xylaria*, *Diaporthe* and *Penicillium* genera are known for their chemical diversity and ability to produce bioactive compounds (Schneider et al. 1996; Oliveira *et al.* 2009; Specian *et al.* 2012; Baraban *et al.* 2013; Lai *et al.* 2013), which demonstrate antipathogen properties *in vitro* (Arnold *et al.* 2003; Thirumalesh *et al.* 2014). Tests for inter-endophyte interactions demonstrated overall positive growth effects in endophytic partners. This suggests that positive interactions between endophytic mutualists within the host plant might occur, which would help to explain their enhanced benefit to the host plant (Afkhami et al. 2014).

Since the endophytic fungi *Micosphaerella*, *Xylaria*, *Diaporthe* and *Penicillium* dominate the endophyte community in *E. coccineum*, and considering their ability to reduce the growth of pathogens *in vitro*, results here provide convincing evidence to suggest that these genera play a role in the protection of *E. coccineum* under natural conditions. Further studies should consider inoculation experiments *in planta* in order to reliably detect positive host plant endophyte effects (Adame-Álvarez *et al.* 2014).

## Conclusions

This study represents the first attempt to link FEF infection intensity and host protection under natural conditions using molecular approaches. Data here showed a relatively high diversity of fungal endophytes associated with leaves of *E. coccineum.* The fungal endophyte community was dominated by just four endophyte genera, which were able to reduce the growth of common pathogens in *vitro*. In addition, colonization by these genera resulted in lower levels of predation of the host plant in natural conditions. These results provide further evidence that colonization by multiple foliar endophytic fungi confers important benefits to host plants in terms of resistance to natural enemies.

## Accession Numbers

Sequence data were submitted to NCBI (accession numbers: KU743942-KU743975).

## Sources of Funding

This study was funded by FONDECYT (Fondo Nacional de Desarrollo Científico y Tecnológico, Chile) Grant no. 11130039.

## Contributions by the Authors

I am the only author of the manuscript.

## Conflicts of Interest Statement

None declared.

## Supplementary Material

Supplementary Data

## Supporting Information

The following additional information is available in the online version of this article —

**Fig. S1**. Distribution of the percentage of leaf damage in 35 individuals (in chronological order) of *Embothrium coocineum* in the Valdivian rainforest.

**Table S1**. Specific primers and probes designed for the four most abundant endophytic fungi (*Xylaria*, *Diaporthe*, *Penicillium* and *Micosphaerella*) isolated from leaves of *Embothrium coccineum*.
