# Supplementary material for: The defensive role of foliar endophytic fungi for a South American tree
Source: AoB Plants. 2016 Aug 2;8:plw050. doi: 10.1093/aobpla/plw050 (PMC4972461; doi:10.1093/aobpla/plw050)
Supplement: Supplementary Data [file supp_8_plw050_index.html]

The defensive role of foliar endophytic fungi for a South American tree — Supplementary Data 

# The defensive role of foliar endophytic fungi for a South American tree

## Supplementary Data

files

- Supplementary Data - zip file
