# Supplementary figures and images for: The defensive role of foliar endophytic fungi for a South American tree
Source: AoB Plants. 2016 Aug 2;8:plw050. doi: 10.1093/aobpla/plw050 (PMC4972461; doi:10.1093/aobpla/plw050)

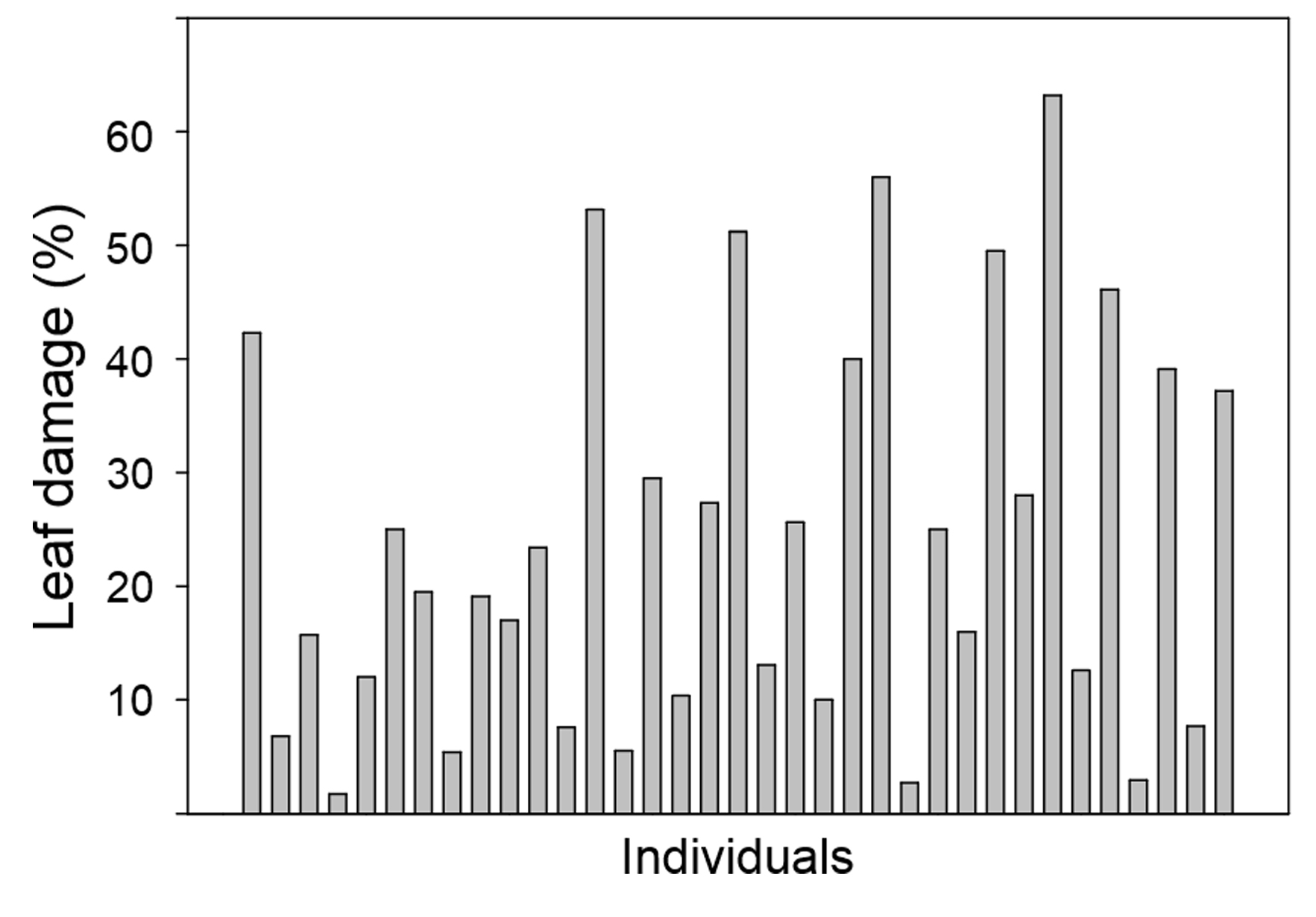

Supplement: Supplementary Data [file supp_plw050_suppl_data.zip › aobplants-16032-s01.jpg]
