# Supplementary material for: The defensive role of foliar endophytic fungi for a South American tree
Source: AoB Plants. 2016 Aug 2;8:plw050. doi: 10.1093/aobpla/plw050 (PMC4972461; doi:10.1093/aobpla/plw050)
Supplement: Supplementary Data [file supp_plw050_suppl_data.zip › aobplants-16032-s02.doc]

**Table S1:** Specific primers and probes designed for the four most abundant endophytic fungi (*Xylaria*, *Diaporthe*, *Penicillium* and *Micosphaerella*) isolated from leaves of *Embothrium coccineum*.

| **Endophytic fungi** | **Primer/Probe name** | **Sequence** | **Modification** |
| --- | --- | --- | --- |
| *Xylaria* | Xy3F | GAG TGT AAT AAC TCC CAA ACC |  |
| *Xylaria* | Xy3R | TTT AGT TAT AGG TTC AGA ATT CAA |  |
| *Xylaria* | Xy3P | ATA CCT CAT GTT GCC TCG GC | 5` 6-FAM-3`TAMRA |
| *Diaporthe* | Di3F | GAC AAC CCT TTG TGA ACT TA |  |
| *Diaporthe* | Di3R | TCA GAG ATT CAG TGT AAA AAC A |  |
| *Diaporthe* | Di3P | CCT ATA CTG TTG CCT CGG CG | 5` 6-FAM-3`TAMRA |
| *Penicillium* | Pe3F | ACC CGT GTT TAT TTT ACC TT |  |
| *Penicillium* | Pe3R | TCA GAC TTC AAT CTT CAG ACA |  |
| *Penicillium* | Pe3P | CCG AAG ACA CCC TCG AAC TC | 5` 6-FAM-3`TAMRA |
| *Mycosphaerella* | My3F | CTG AGC AGT TCG TCT CTA AG |  |
| *Mycosphaerella* | My3R | CTT AAG TTC AGC GGG TAT C |  |
| *Mycosphaerella* | My3P | ACG GCT TTT GGC CGT TAA AT | 5` 6-FAM-3`TAMRA |
